# Effect of an NHE3 inhibitor in combination with an NPT2b inhibitor on gastrointestinal phosphate absorption in Rodent models

**DOI:** 10.1371/journal.pone.0292091

**Published:** 2024-01-26

**Authors:** Xiaojun Wang, Xiaohong Yu, Kostas Gavardinas, Asim Dey, Hong Y. Zhang, Gina Porter, Leah Porras, Lan Yu, Haihong Guo, Charles A. Reidy, Joseph V. Haas, Yanping Xu, Mark C. Kowala, Prabhakar K. Jadhav, John R. Wetterau

**Affiliations:** Lilly Research Laboratories, Eli Lilly and Company, Indianapolis, Indiana, United States of America; Anatomy, SWITZERLAND

## Abstract

Many of the pathological consequences of chronic kidney disease can be attributed to an elevation in serum phosphate levels. Current therapies focused on decreasing intestinal phosphate absorption to treat hyperphosphatemia are inadequate. The most effective therapeutic strategy may be to target multiple absorptive pathways. In this study, the ability of a novel inhibitor of the intestinal sodium hydrogen exchanger 3 (NHE3), LY3304000, which inhibits paracellular, diffusional uptake of phosphate, to work in combination with an inhibitor of the active transporter, sodium dependent phosphate cotransporter 2b (NPT2b), LY3358966, was explored. LY3304000 modestly inhibited the acute uptake of phosphate into plasma of rats, while surprisingly, it doubled the rate of phosphate uptake in mice, an animal model dominated by NPT2b mediated acute phosphate uptake. In rats, LY3004000 and LY3358966 work in concert to inhibit acute phosphate uptake. On top of LY3358966, LY3304000 further decreased the acute uptake of phosphate into plasma. Studies measuring the recovery of radiolabeled phosphate in the intestine demonstrated LY3304000 and LY3358966 synergistically inhibited the absorption of phosphate in rats. We hypothesize the synergism is because the NHE3 inhibitor, LY3304000, has two opposing effects on intestinal phosphate absorption in rats, first it decreases diffusion mediated paracellular phosphate absorption, while second, it simultaneously increases phosphate absorption through the NPT2b pathway. NHE3 inhibition decreases proton export from enterocytes and raises the cell surface pH. In vitro, NPT2b mediated phosphate transport is increased at higher pHs. The increased NPT2b mediated transport induced by NHE3 inhibition is masked in rats which have relatively low levels of NPT2b mediated phosphate transport, by the more robust inhibition of diffusion mediated phosphate absorption. Thus, the inhibition of NPT2b mediated phosphate transport in rats in the presence of NHE3 inhibition has an effect that exceeds its effect in the absence of NHE3 inhibition, leading to the observed synergism on phosphate absorption between NPT2b and NHE3 inhibition.

## Introduction

Patients with chronic kidney disease (CKD) and end stage renal disease have an impaired ability to excrete phosphate in urine, which leads to hyperphosphatemia. The increased serum phosphate may promote vascular and heart valve calcification, and calcium phosphate deposits in various organs including heart and kidney [1–5]. To compensate for the increase in serum phosphate, FGF23 and PTH are increased, which promotes phosphate excretion by the kidney [6]. In addition, FGF23 lowers 1,25-dihydroxyvitamin D levels which results in reduced intestinal phosphate absorption [7]. However, the FGF23 and PTH may promote cardiac hypertrophy [8, 9] that can contribute to the increase in arrhythmias and sudden cardiac death observed in patients with renal disease [10, 11]. The changes in FGF23, PTH and 1,25-dihydroxyvitamin D also promote CKD mineral bone disease [12].

Current strategies to treat hyperphosphatemia focus on reducing intestinal phosphate absorption to balance the decrease in renal phosphate excretion. Intestinal phosphate is absorbed through paracellular diffusion and active transporter mediated transcellular transport [13]. Currently available approaches to reduce intestinal phosphate absorption include restricting dietary phosphate content and the use of various phosphate binders. Both approaches reduce the free phosphate levels in the intestinal lumen and decrease diffusional phosphate uptake. However, these approaches have limitations, including the high phosphate content of a typical Western diet, limited binding capacity of phosphate binders, and poor patient compliance. As a result, the majority of dialysis patients are unable to maintain serum phosphate at desired levels [13].

The inability to manage serum phosphate levels has led to a search for newer, more effective ways to inhibit intestinal phosphate absorption. More recently, a gut restricted sodium-hydrogen exchanger 3 (NHE3) inhibitor, tenapanor, was developed to treat hyperphosphatemia [14, 15]. Inhibition of NHE3 on the apical surface of intestinal epithelial cells decreases the paracellular diffusion mediated uptake of phosphate [16]. Although tenapanor has been extensively studied in the clinic, it is yet to be FDA approved for the treatment of hyperphosphatemia, with limited efficacy being cited for the delay. Two inhibitors of the sodium dependent phosphate cotransporter 2b, NPT2b, have progressed to the clinic, but had minimal or no effect on intestinal phosphate absorption [17, 18]. In contrast, a pan NPT2b, Pit-1 and Pit-2 inhibitor did inhibit phosphate absorption in a small clinical trial in ESRD patients [19].

Despite the available therapies and drug candidates in development, there is still a need for more effective means to control phosphate levels in advanced renal disease. The multiple complementary mechanisms by which phosphate can be absorbed in the intestine makes it a challenge for any one targeted therapy to provide robust efficacy. When one route of absorption is decreased or blocked, the phosphate could be absorbed through another path. In addition, the other pathways could be upregulated to compensate for the loss of one pathway. An example of this is the well-established upregulation of NPT2b when dietary phosphate is restricted [20].

An ideal therapy to decrease intestinal phosphate absorption would decrease both active and passive diffusional transports. To explore this, we previously tested the ability of a potent NPT2b inhibitor to work alone and in combination with the phosphate binder, sevelamer, in two preclinical animal models [21]. While NPT2b had a prominent role in the acute uptake of phosphate into plasma of mice, NPT2b inhibition minimally affected phosphate absorption over time, highlighting the ability of other phosphate absorption pathways to compensate for the loss of NPT2b activity. When the NPT2b inhibitor was used in combination with the phosphate binder sevelamer to further inhibit the diffusion uptake pathway, the inhibition of phosphate absorption was still minimal, suggesting an NPT2b inhibitor/sevelamer combination is not ideal.

The goal of this study was to characterize the ability of a novel NHE3 inhibitor, LY3304000, to reduce intestinal phosphate uptake in mice and rats, and further test its ability to work in combination with a previously characterized NPT2b inhibitor, LY3358966. An NHE3 inhibitor, like sevelamer, inhibits the diffusional uptake of intestinal phosphate. In contrast to the phosphate binding mechanism employed by a phosphate binder, NHE3 inhibition is hypothesized to modulate the conformation of intestine tight junctions which control paracellular phosphate diffusion [16].

## Materials and methods

### Generation of a sodium hydrogen exchanger (NHE) deficient cell line

Sodium hydrogen exchanger activity specific to NHE3 was achieved by overexpressing human NHE3 in an endogenously NHE-deficient cell line generated by chemical-induced mutagenesis [22]. After the mutagenesis, NHE-deficient cells were selected by loading lithium into the cells and then exposing them to an acidic extracellular environment. Under these conditions, NHE exchanges intracellular lithium for extracellular protons, thus only NHE-deficient cells can avoid a toxic intracellular acidosis and survive. Dede cells (ATCC ccl-39), grown to 50% confluence in McCoy’s 5A medium (GE Healthcare) with 10% FBS and antibiotic/antimycotic solution (GE Healthcare) in a T75 flask, were treated with 500 μg/mL of EMS (ethyl methanesulfonate, Sigma-Aldrich) in medium for 20 hours for mutagenesis and washed twice with phosphate buffered saline (PBS). The cells were then trypsinized, plated in two T175 flasks and cultured in fresh medium without EMS for 2 days. The cells were again trypsinized, plated in four T175 flasks and cultured in the same medium overnight. The cells were incubated with 20 mL per flask of LiCl Solution (130 mM LiCl, 5 mM KCl, 1 mM MgSO_4_, 2 mM CaCl_2_, 5mM glucose and 20 mM HEPES-Tris, pH 7.4) for 2 hours at 37°C, then washed once with 10 mL per flask of the LiCl Solution. The cells were then incubated with 20 mL per flask of Choline Chloride Solution (130 mM choline chloride, 5 mM KCl, 1 mM MgSO_4_, 2 mM CaCl_2_, and 20 mM 2-(N-morpholino) ethanesulfonic acid-Tris, pH 5.5) for 30 minutes, then washed once with 10 mL per flask of the Choline Chloride Solution. The cells were washed with PBS twice, trypsinized and plated in two T175 flasks. After a week of culture in medium, the surviving cells grew to 90% confluence and were subjected to a second cycle of selection following the above protocol. The single-clone colonies of surviving cells, isolated by serial dilutions, were trypsinized and plated in T25 flasks. After reaching full confluence, the surviving cells were subjected to a third cycle of selection following the above protocol. The surviving cells were then expanded and tested for deficiency of NHE activity. NHD C8 is a single clone cell line that we generated which was sodium hydrogen exchanger (NHE) deficient.

### Generation of human NHE3 over-expressing cells

The human NHE3 cDNA (accession number: NM_004174.2, GeneScript) with a myc-tag was cloned into a pcDNA3.1 vector. NHD C8 cells were maintained in McCoy’s 5A medium and transfected with the cDNA construct containing human NHE3 using Lipofectamine reagent (ThermoFisher Scientific). Individual neomycin-resistant cell clones were selected in medium containing 400 μg/mL G418 (ThermoFisher Scientific) and tested for sodium-hydrogen exchanger (NHE) activity. The cell clone that displayed the highest NHE activity was expanded and used for the development of the NHE activity assay.

### NHE3 activity assay

The NHE activity assay is based on the principle that NHE mediates extracellular sodium and intracellular hydrogen exchange after an intracellular acid overload. The intracellular pH change is then measured by an intracellular pH-sensitive dye, 2’,7’-Bis-(2-carboxyethyl)-5(and-6)-carboxyfluorescein, acetoxymethyl ester (BCECF-AM, ThermoFisher Scientific) upon reintroduction of sodium. NHE3 overexpressing NHD C8 cells were dispersed into 96-well poly-D-lysine plates (Corning) at 30,000 cells per well. The cells were incubated at 37°C plus 5% CO_2_ for 24 hours. Cell culture medium was aspirated and cells were washed with 100 μL of NaCl-HEPES Solution (100 mM NaCl, 10 mM glucose, 5 mM KCl, 2 mM CaCl_2_, 0.1% BSA, 1 mM MgCl_2_, 50 mM HEPES, pH 7.0) twice, then cells were incubated with 100 μL of 5 μM BCECF-AM in NH_4_Cl/pluronicF-127/probenecid Solution (130 mM NH_4_Cl, 5 mM KCl, 2 mM CaCl_2_, 1 mM MgCl_2_, 0.1% BSA, 0.0625% pluronic F-127, 2 mM probenecid, 20 mM HEPES, pH 7.0) for 60 minutes at room temperature. The cells were then washed with 100 μL of ammonium free, sodium free, HEPES/0.1% BSA solution (100 mM choline chloride, 10 mM glucose, 5 mM KCl, 2 mM CaCl_2_, 1 mM MgCl_2_, 0.1% BSA, 50 mM HEPES, pH 7.0) twice. Following the addition of 86 μL of ammonium free, sodium free, HEPES/0.1% BSA solution containing 30 μM amiloride, compounds or controls in the appropriate wells, the NHE activity was initiated by the addition of 14 μL of 1 M NaCl except the baseline control wells with 14 μL of 1M Choline Chloride. The final concentration of sodium was 140 mM. The plate was immediately read for fluorescence at the excitation wavelength of 505 nm and the emission wavelength of 550 nm using a kinetic method by Molecular Device, SpectraMax i3. Percentage of inhibition at each concentration tested was calculated relative to NHE activity with 1% DMSO as 0% inhibition and NHE activity with a saturating concentration of a standard inhibitor as 100% inhibition. A 9-concentration response curve from 1 μM to 0.152 nM was fitted to a 4-parameter model using Prism to determine the half maximal inhibitory concentration (IC_50_).

### NPT2b, Pit-2 and SGLT1 activity assays

Generation of inducible human, mouse, and rat NPT2b overexpressing T-Rex^™^ Chinese Hamster Ovary (CHO, Thermo Fisher Scientific) cells and the NPT2b activity assay were described previously [21]. Briefly, cells were plated in 96-well CytoStar-T^®^ scintillating microplates and were incubated overnight in medium plus 100 ng/mL of tetracycline to induce the expression of human NPT2b. The cells were then washed 3 times with 200 μL Assay Buffer (137 mM NaCl, 5.4 mM KCl, 2.8 mM CaCl_2_, 1.2 mM MgSO_4_, and 14 mM Tris-HCl buffer, pH 7.5). LY3304000 serially diluted 1-to-3 in DMSO was added to cells with Assay Buffer. An equal volume of H_3_^33^PO_4_ (PerkinElmer) in Assay Buffer supplemented with 5 μM potassium phosphate was then added to initiate radiolabeled phosphate (^33^P-phosphate) uptake. The final phosphate concentration was 2.5 μM. Following a 60-minute incubation at room temperature, an equal volume of Assay Buffer containing 400 μM phloretin was added to stop ^33^P-phosphate uptake. The plate was immediately read on a Wallac MicroBeta Trilux liquid scintillation counter. Percent inhibition at all concentrations tested was calculated, and IC_50_ values were then determined using a 4-parameter logistic curve fitting equation.

To determine the effect of pH on NPT2b activity in vitro, ^33^P-phosphate uptake was measured in human, mouse, and rat NPT2b overexpressing T-Rex^™^ CHO cells in sodium or choline containing Assay Buffer with pH adjusted to 5.5 and 6.5 by MES buffer, or 7.5 and 8.5 by Tris-HCl buffer (137 mM NaCl/CholineCl, 5.4 mM KCl, 2.8 mM CaCl_2_, 1.2 mM MgSO_4_, and 30 mM MES buffer, pH 5.5 or 6.5 and 137 mM NaCl/CholineCl, 5.4 mM KCl, 2.8 mM CaCl_2_, 1.2 mM MgSO_4_, and 30 mM Tris-HCl buffer, pH 7.5 or 8.5) as described above.

Generation of constitutive human Pit-2 and SGLT1 overexpressing CHO cells and activity assays measuring the uptake of ^33^P phosphate for 45 minutes at 20°C for Pit-2 and ^14^C-α-methyl-D-glucopyranoside for 120 minutes at 37°C for SGLT1, were also described previously [21].

### Formulation

The detail synthesis LY3304000, (Z)-[[4-[[(Z)-N’-carbamoyl-N-[2-[2-[2-[[3-[(4S)-6,8-dichloro-2-methyl-3,4-dihydro-1H-isoquinolin-4-yl]phenyl]sulfonylamino]ethoxy]ethoxy]ethyl]carbamimidoyl]amino]butylamino]-[2-[2-[2-[[3-[(4S)-6,8-dichloro-2-methyl-3,4-dihydro-1H-isoquinolin-4-yl]phenyl]sulfonylamino]ethoxy]ethoxy]ethylamino]methylene]urea dihydrochloride, is described in Scheme 1 in the S1 File. To prepare a dosing solution, LY3304000 was dissolved in 1% HEC vehicle consisting of 1% hydroxyethylcellulose, 0.25% polysorbate 80, 0.05% antifoam in purified water.

LY3358966 was synthesized as previously described [21]. The free base of LY3358966 was formulated in poly-1-vinylpyrrolidone-co-vinyl acetate (PVP-VA) in methanol using spray dried solid dispersion (SDD) as described previously [21]. Briefly, two mole equivalents of NaOH were added to the slurry containing 30% LY3358966 and 70% PVP-VA, which was then bath sonicated until a clear yellow solution was formed. The solution was slowly pumped into a spray dryer with a stream of hot nitrogen resulting in a solid powder that was collected and further dried in a vacuum oven at 50°C. To prepare a dosing solution, the LY3358966 SDD was dissolved in water to achieve the desired concentration of active pharmacological ingredient (API). This amorphous solid dispersion formulation was developed to optimize the dissolution and solubility properties of LY3358966. Doses are expressed as active pharmaceutical ingredient (API) in below studies.

### In vivo studies

All animal procedures were approved by and conducted in accordance with the Eli Lilly and Covance Institutional Animal Care and Use Committee guidelines. Male Sprague Dawley (SD) rats, approximately 160 to 180 grams, were obtained from Charles River Laboratories. Male C57Bl/6 mice, approximately 8 to 9 weeks old, weighing 22 to 28 grams, were obtained from Taconic BioSciences, Inc. Animals were acclimated to single housing for a minimum of 3–4 days and fed with Teklad Global 14% Protein Rodent Maintenance Diet (Envigo) containing 0.6% of phosphorus (0.3% non-phytate phosphorus) with free access to tap water. Before initiating the studies, the animals were randomized into groups with equal mean body weight using a Block Randomization Tool (BRAT).

### Effect of LY3304000 on acute phosphate uptake into plasma in rats

A dosing solution for a 10 mg/kg dose of LY3304000 using a 10 mL/kg dosing volume was made in 1% HEC vehicle. The subsequent dosing solutions for 3, 1, 0.3, 0.1, 0.03, 0.01, 0.003, and 0.001 mg/kg were prepared by serial dilutions with HEC. A Radiolabeled Phosphate Dosing Solution was made in a 16.25 mM Na_2_HPO_4_, 0.9% saline, pH 7.4, solution with the addition of about 2.5 μCi/mL radioactive phosphate (H_3_^33^PO_4_, PerkinElmer) and filtered before dosing. Following 12-hour fasting with access to water, male SD rats were orally dosed at 10 mL/kg with either vehicle or varying doses of LY3304000. Fifteen minutes later, 2 mL of the Radiolabeled Phosphate Dosing Solution was dosed by oral gavage. This dose of phosphate was chosen because it approximates the rat equivalent to the amount of bioavailable phosphate in a human meal, 4 mg/kg phosphorus, or 320 mg phosphorus for an 80 kg person on a phosphate restricted diet. Fifteen minutes later, blood was collected by cardiac stick, and plasma was prepared. Radioactivity (dpm) in 50 μL plasma was measured by scintillation counting. To determine the total radioactivity dosed to each rat, the radioactivity in two 10-μL samples of the Radiolabeled Phosphate Dosing Solution was measured by scintillation counting. To calculate the total radioactivity recovered in plasma, it was assumed that total blood volume constitutes about 60% of the body weight and plasma constitutes about 55% of the total blood. The mole amount of phosphate uptake was calculated as the percentage of administered radiolabeled phosphate recovered in the plasma of vehicle or LY3304000 treated rats multiplied by total phosphate amount in the radiolabeled phosphate dosing solution. ED_50_ values were then determined with a 4-parameter logistic curve fitting model using GraphPad Prism.

A 15-minute time point, post phosphate dosing, was chosen for collecting blood in the acute phosphate uptake studies in rats and mice (see below) to capture the initial rate of phosphate absorption. We wanted to avoid measurements at longer time points when plasma radiolabeled phosphate appears to be at a steady state [23, 24].

### Effect of LY3304000 on acute phosphate uptake in mice

LY3304000 dosing solution and Radioactive Phosphate Dosing Solution were made as described above. Following 12-hour fasting with access to water, male C57Bl/6 mice were dosed orally at 10 mL/kg with either vehicle or varying doses of LY3304000. Fifteen minutes later, 200 μL of the Radiolabeled Phosphate Dosing Solution was dosed by oral gavage. Fifteen minutes later, blood was collected by cardiac stick, and plasma was prepared. The measurement of acute phosphate uptake was determined as described above for rats.

### Effect of LY3304000 on sodium and phosphate excretion in urine in rats

To assess sodium and phosphate absorption in rats, we took advantage of the increase in urinary sodium and phosphate excretion following an oral bolus of sodium phosphate. Thus, an LY3304000 dependent decrease in urinary sodium and phosphate excretion following an oral bolus dose of sodium phosphate is an indirect measurement of the ability of LY3304000 to inhibit sodium and phosphate absorption in intestine.

A dosing solution of 3 mg/kg LY3304000 for a 10 mL/kg dose volume was formulated in 1% HEC. The subsequent dosing solutions of 1, 0.3, 0.1, 0.03, 0.01, 0.003, and 0.001 mg/kg LY3304000 were prepared by serial dilutions with 1% HEC. An NaH_2_PO_4_ dosing solution at 690 mg/kg with a 20 mL/kg dosing volume was made in sterile water. Male SD rats were fasted for 4 hours with access to water and then orally dosed at 10 mL/kg with 1% HEC vehicle or varying dose of LY3304000 at 0.001, 0.003, 0.01, 0.03, 0.1, 0.3, 1, and 3 mg/kg. In this and subsequent studies where phosphate absorption is measured, a 4 hour fast was used which is less disruptive to the animal’s normal physiology than the 12 hours fast used to measure acute phosphate uptake into plasma. Fifteen minutes later, rats were dosed with either sterile water or NaH_2_PO_4_ solution, and then immediately transferred to metabolic cages. Urine samples were collected from the metabolic cages at 4 hours post sterile water or NaH_2_PO_4_ dose. The net urine volume was recorded. Urinary sodium, creatinine, and phosphorus (as a measure of phosphate) were determined using a clinical biochemistry analyzer. The urinary sodium-to-creatinine and phosphorus-to-creatinine ratios were calculated and compared between vehicle and LY3304000 treated groups. The ratios of urinary-to-dietary phosphate (measured as phosphorus) and urinary-to-dietary sodium in urine were compared between vehicle and LY3304000 treated groups.

### Effect of LY3304000 on sodium and phosphate excretion in urine in mice

The effect of LY3304000 on sodium and phosphate excretion in urine was also assessed in mice. A dosing solution of 3 mg/kg LY3304000 for a 10 mL/kg dose volume was formulated in 1% HEC. An NaH_2_PO_4_ dosing solution at a 690 mg/kg with a 40 mL/kg dosing volume was made in sterile water. Male C57Bl/6 mice were fasted for 4 hours with access to water and then orally dosed at 10 mL/kg with 1% HEC vehicle or 3 mg/kg LY3304000. Fifteen minutes later, mice were orally dosed with either sterile water or NaH_2_PO_4_ solution, and then immediately transferred to a mouse holder on a 96-well plate for spot urine collection. Urine samples were collected for 4 hours post sterile water or NaH_2_PO_4_ dose. Urinary sodium, creatinine, and phosphorus (as a measure of phosphate) were determined using a clinical biochemistry analyzer. The urinary sodium-to-creatinine ratio was calculated and compared between vehicle and LY3304000 treated groups.

### Effect of LY3304000 used in combination with LY3358966 on acute phosphate uptake in rats

NHE3 inhibitor LY3304000 and NPT2b inhibitor LY3358966 SDD were used in combination to assess their effect on phosphate absorption in rats. To make the LY3358966 dosing solution, an appropriate amount of LY3358966 SDD was weighed for a 1.2 mg/kg API dosing solution with 10 mL/kg dosing volume and dissolved in water. To make the LY3304000 dosing solution, an appropriate amount of LY3304000 was weighed for a 10 mg/kg dose with 10 mL/kg dosing volume and dissolved in 1% HEC vehicle. The subsequent dosing solutions for 3, 1, 0.3, 0.1, 0.03, 0.01, 0.003, and 0.001 mg/kg LY3304000 were prepared by serial dilutions with 1% HEC.

Following 12-hour fasting, male SD rats were orally dosed at 10 mL/kg with 1% HEC vehicle, 1.2 mg/kg LY3358966 or increasing doses of LY3304000 with a fixed, 1.2 mg/kg dose of LY3358966. In the vehicle group, the animals were dosed first with 1% HEC immediately followed by water. In the LY3358966 alone group, the animals were first dosed with 1% HEC immediately followed by LY3358966. In the combination groups, the animals were dosed first with LY3304000 immediately followed by LY3358966. Fifteen minutes later, 2 mL Radiolabeled Phosphate Dosing Solution was orally dosed. Fifteen minutes later, blood was collected by cardiac stick, and plasma was prepared. Radioactivity (dpm) in 50-μL plasma was measured by scintillation counting. To measure the total radioactivity dosed to each rat, the radioactivity in two 10-μL samples of the Radiolabeled Phosphate Dosing Solutions was measured by scintillation counting. The mole amount of phosphate uptake was calculated as described above to determine acute phosphate uptake.

### In vivo inhibition of phosphate absorption by LY3304000, LY3358966, and an LY3304000/LY3358966 combination in rats

We also evaluated the effect of LY3304000 and LY3358966 in combination on phosphate absorption in rats by measuring radioactive phosphate recovered in either plasma or the gastrointestinal (GI) tract. In this combination study, two vehicles were used. One is 1% HEC vehicle for making LY3304000 dosing solution, another is 0.46% PVP-VA vehicle contained in LY3358966 SDD. To make the LY3358966 dosing solution, an appropriate amount of LY3358966-SDD was weighed for a 10 mg/kg API dosing solution with 5 mL/kg dosing volume and dissolved in water. To make the LY3304000 dosing solution, an appropriate amount of LY3304000 was weighed for a 0.4 mg/kg dosing solution with 5 mL/kg dosing volume and dissolved in 1% HEC. The Radiolabeled Phosphate Dosing Solutions was made as described above. Following 4-hour fasting with access to water, male SD rats were orally dosed at 10 mL/kg with either two vehicles, an LY3358966 dosing solution and 1% HEC vehicle, an LY3304000 dosing solution and 0.46% PVP-VA vehicle, or an LY3304000 and LY3358966 dosing solutions, combined 1:1 by volume. Fifteen minutes later, Radiolabeled Phosphate Dosing Solution was orally dosed in a 2 mL volume. Fifteen minutes after the radiolabeled phosphate dose, blood was collected by retro orbital bleeding, and plasma was prepared. Radioactivity (dpm) in 20-μL plasma was measured by scintillation counting. The mole amount of phosphate uptake in the plasma was calculated as described above. Four hours post radioactive phosphate dose, the animals were sacrificed by asphyxiation with CO_2_ followed by cervical dislocation and then the stomach, small intestine, large intestine, and feces were collected and weighed. The tissues and feces were digested in 20 mL or 10 mL of 1 N NaOH, respectively, overnight at 37°C. A 100 μL sample of the homogenates were dispensed into scintillation vials and mixed with 10 mL of Optiphase Supermax scintillation fluid (PerkinElmer), and radioactivity (dpm) was measured by scintillation counting. Very little radioactivity was detected in the feces at 4 hours post radioactive phosphate dose. The percentage of radiolabeled phosphate recovered in the gastrointestinal tissue was defined as radioactivity (dpm) recovered in each fraction compared to the amount of radioactivity (dpm) administered.

The mole amount of phosphate uptake into plasma in vehicle and LY3358966 and LY3304000 alone groups were compared to the LY3358966 and LY3304000 combination group using One-way ANOVA with Dunnett’s multiple comparisons.

Percent recovery of ^33^P phosphate in the rat gastrointestinal tract was analyzed by two-way analysis of variance (ANOVA) in JMP 12.1 to test for additivity of the effects of LY3304000 and LY3358966 on inhibiting phosphate absorption. A significant (p<0.05) test of the LY3304000 and LY3358966 interaction effect would indicate a synergistic relationship between the compounds at the doses tested in this study, suggesting the inhibitory effect of the combination is greater than the sum of the individual compound effects. The effect of the combination of LY3304000 and LY3358966 on inhibition of phosphate absorption was also compared to the individual compound effects by the Tukey’s HSD method to test whether the combination has significantly (p<0.05) higher inhibition of phosphate absorption than either compound alone.

## Results

### In vitro activity of LY3304000

**Fig 1:**
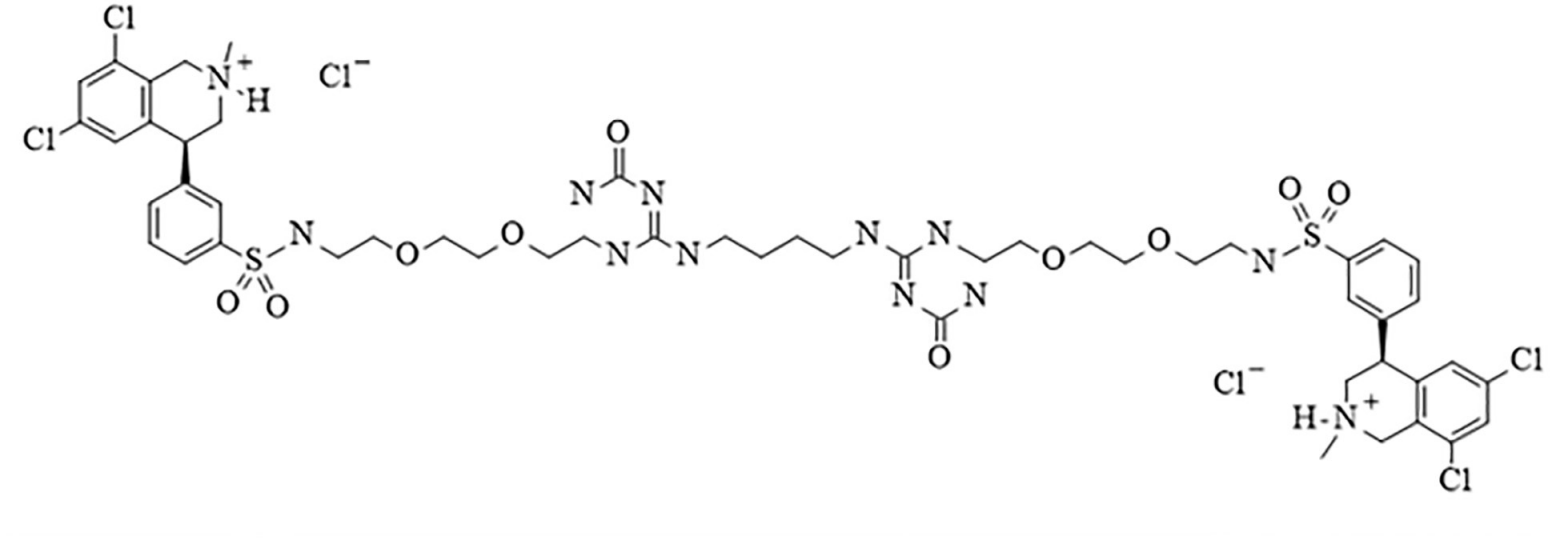
Chemical structure of LY3304000.

**Fig 2:**
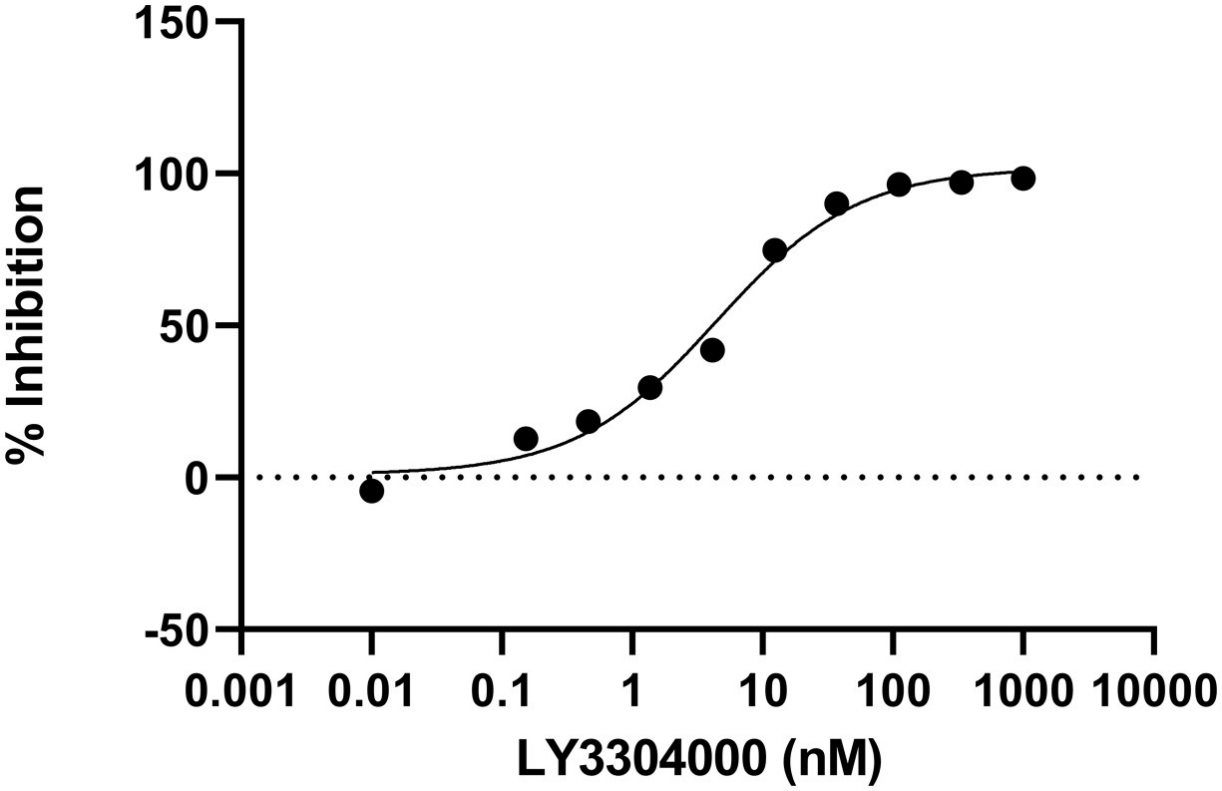
In vitro human NHE3 activity of LY3304000.

The NHE3 inhibitor, LY3304000 (Fig 1 and Scheme 1 in S1 File) was discovered by a medicinal chemistry campaign. The molecule was optimized to minimize its absorption in the intestine (see S1 File) and for its ability to inhibit NHE3. To assess its inhibition on NHE3 activity, Dede cells were made NHE deficient by chemical mutagenesis, then used to make a human NHE3 overexpressing cell line for assessing NHE3 activity. LY3304000 is a potent NHE3 inhibitor with an IC_50_ of 5.8 nM for human NHE3 (Fig 2 and Table 1). To explore the selectivity of LY3304000, it was also tested for the ability to inhibit two sodium dependent phosphate transporters found in the intestine, NPT2b and Pit-2, and the sodium dependent glucose transporter 1, SGLT1. LY3304000 is highly selective for inhibition of NHE3 versus NPT2b, Pit-2 and SGLT1 (Table 1).

**Table 1:** Summary of In Vitro Inhibition of Human NHE3 (A), Human NPT2b, Pit2 and SGLT1 (B) by LY3304000.

**A**.			
**NHE3 Assays**	**IC**_**50**_ **, nM** ^1^	**SEM** ^2^	**n** ^3^
**Human NHE3**	5.76	0.72	7
**B**.			
**Assays**	**IC**_**50**_ **, μM** ^1^	**SEM** ^2^	**n** ^3^
**Human NPT2b**	58.4	4.57	3
**Human Pit-2**	>100	NA	1
**Human SGLT1**	6.60	2.10	4

^1^ IC_50_ values represent geometric mean of individual IC_50_.

^2^ SEM represents standard error of geometric mean in molar units.

^3^ n represents numbers of individual studies on different days.

A representative concentration-response curve of LY3304000 in a human NHE3 activity assay is shown.

Percentage of inhibition at each concentration tested was calculated relative to NHE activity with 1% DMSO as 0% inhibition and NHE activity with a saturating concentration of a standard inhibitor as 100% inhibition. A 9-concentration response curve from 1 μM to 0.152 nM was fitted to a 4-parameter model using Prism to determine the half maximal inhibitory concentration (IC_50_). For the purpose of curve fitting, no compound was set to a concentration of 0.01 nM. The slope of the curve obtained from analysis is 0.8.

### In vivo inhibition of NHE3 in mice and rats

Following an oral bolus of an NaH_2_PO_4_ solution to mice or rats, there is an increase in urinary sodium. At a 3 mg/kg dose, LY3304000 suppressed the increase in sodium recovered in urine (Table 2) of mice and rats, indicating the NHE3 inhibitor, LY3304000, effectively inhibits intestinal sodium absorption in vivo. The decreased urinary sodium was attributed to the effects of the compound in the intestine and not the kidney where NHE3 is also found at high levels because LY3304000 could not be detected in plasma following oral dosing. In addition, inhibition of renal NHE3 would increase urinary sodium, not decrease it.

**Table 2:** Effect of LY3304000 on sodium excreted in urine.

Species	Mice			Rats		
Groups	Ratio (mg/mg)^1^	n	*p*-value^3^	Ratio (mg/mg)^1^	n	*p*-value^3^
**Water**	10.8 ± 1.1^2^	8	< 0.0001	3.9 ± 0.7	8	0.0018
**Sodium bolus**	28.9 ± 4.0	8	1.0	10.2 ± 1.1	8	1.0
**Sodium bolus plus 3 mg/kg LY3304000**	14.6 ± 1.7	8	0.0001	1.0 ± 0.4	6	0.0098

^1^ Urinary sodium-to-creatinine ratios (mg/mg) for mice and rats. Data are presented as mean ± SEM.

^2^ Urinary sodium in control mice administered vehicle and water is less than the detectable limit, 20 mmol/L. Thus, the urinary sodium-to-creatinine ratio in this group is less than 10.8.

^3^ *p*-Value was determined by a One-way ANOVA with a Dunnett’s comparison to the group of Sodium bolus using JMP.

### Effect of LY3304000 on acute phosphate uptake into plasma of mice

**Fig 3:**
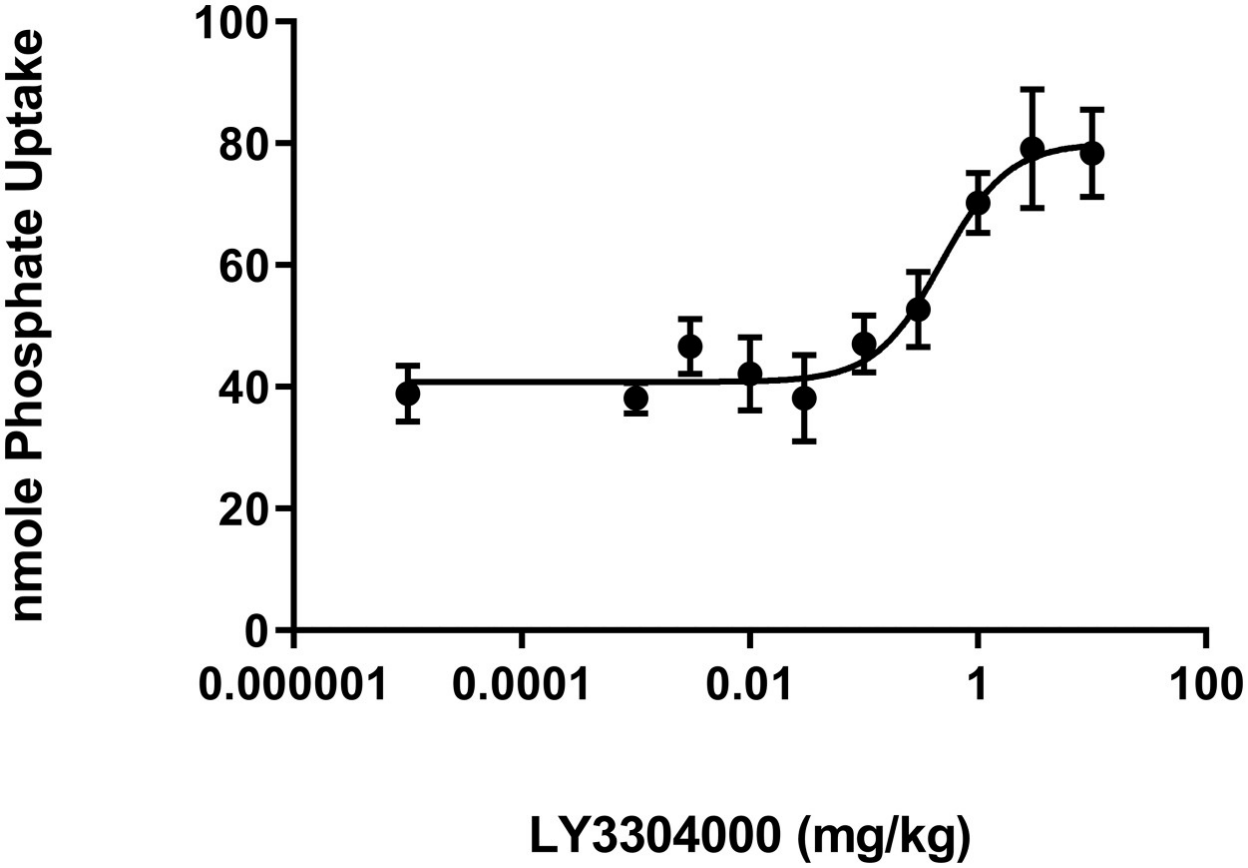
Effect of LY3304000 on acute phosphate uptake in mice.

Fasted C57Bl/6 mice were administered vehicle control or varying doses of LY3304000. Fifteen minutes later, they were dosed with radiolabeled phosphate. Another fifteen minutes later, blood was collected, radioactivity in the plasma was measured and the total amount of ^33^P-phosphate uptake into plasma calculated (Fig 3). In contrast to what has been reported in humans and rats [25, 26], LY3304000 dose-dependently increased phosphate uptake with an ED_50_ of 0.48 mg/kg. Thirty-nine nmoles of radiolabeled phosphate were recovered in plasma in vehicle treated animals, while in LY3304000 treated animals, at the highest doses tested, this was doubled to 78 nmoles.

The absorption of radiolabeled phosphate measured at 15 minutes post dose was determined in mice. The mole amount of total phosphate (Pi) uptake was calculated as the percentage of administered radiolabeled phosphate recovered in the plasma multiplied the total phosphate in the Radiolabeled Phosphate Dosing Solution. Results are presented as mean ± SEM with animal numbers equal to 9 in the vehicle control group and 6 or 7 in LY3304000 treated groups. The curve was fitted with nonlinear regression with variable slope using GraphPad Prism. The slope of the curve obtained from analysis is 1.49. For the purpose of curve fitting, the vehicle was set to a dose of 0.00001 mg/kg. The ED_50_ value was calculated to be 0.479 mg/kg with E_max_ value of 80.01 nmole Pi update.

### Effect of LY3304000 on acute phosphate uptake into plasma of rats

**Fig 4:**
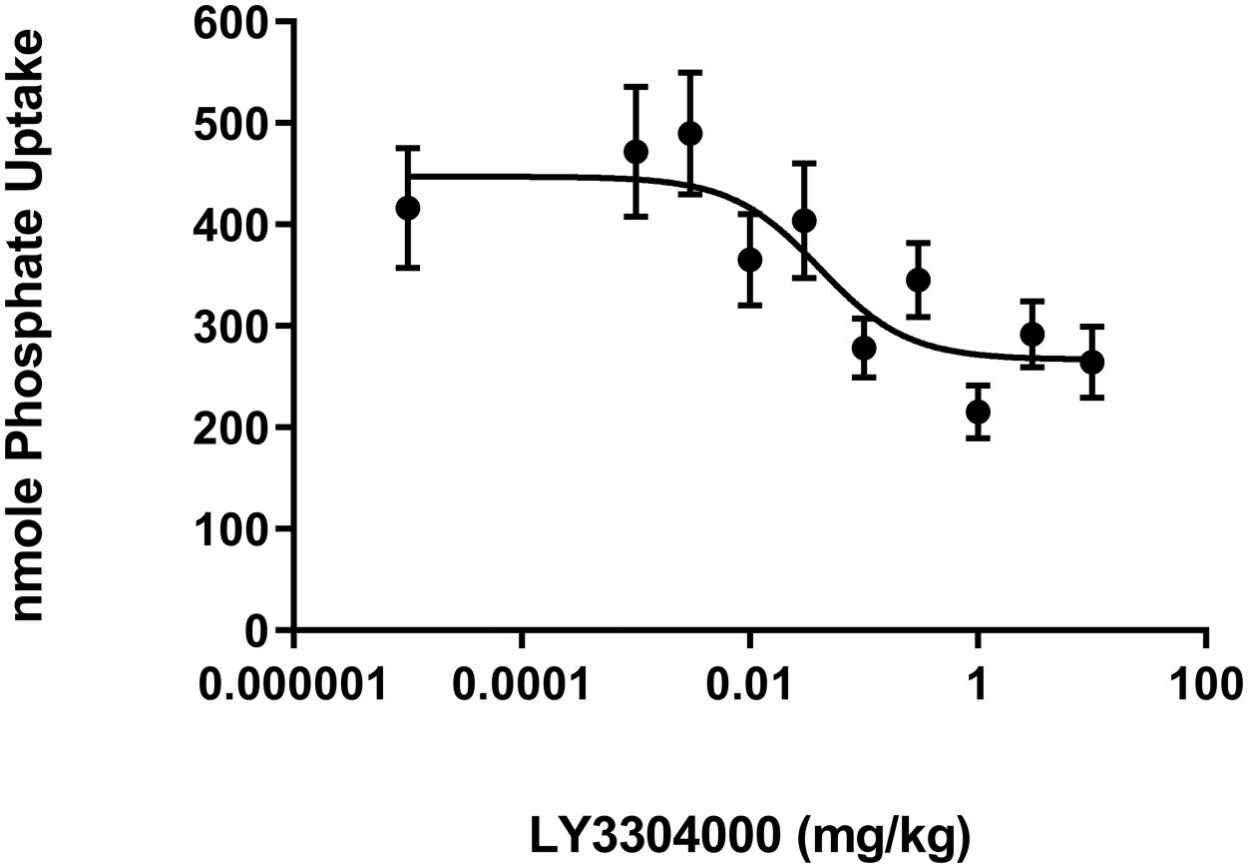
Effect of LY3304000 on acute phosphate uptake in rats.

The effect of LY3304000 on acute radiolabeled phosphate uptake into plasma was measured in fasted male Sprague Dawley (SD) rats using the same protocol design used in mice. In the vehicle group, 416 nmoles of radiolabeled phosphate in radiolabeled phosphate dosing solution were recovered in plasma. LY3304000 dose-dependently decreased phosphate uptake with an ED50 of 0.04 mg/kg (Fig 4). The maximal determined decrease was to 264 nmoles of radiolabeled phosphate, or 37% inhibition.

The absorption of radiolabeled phosphate measured at 15 minutes post dose was determined in rats. The mole amount of total phosphate (Pi) uptake was calculated as the percentage of administered radiolabeled phosphate recovered in the plasma multiplied the total phosphate in the Radiolabeled Phosphate Dosing Solution. Results are presented as mean ± SEM with animal numbers equal to 9 in the vehicle control group and 6 or 7 in LY3304000 treated groups. The curve was fitted with nonlinear regression with variable slope using GraphPad Prism. The slope of the curve obtained from analysis is 1.12. For the purpose of curve fitting, the vehicle was set to a dose of 0.00001 mg/kg. The ED_50_ value was calculated to be 0.041 mg/kg with E_max_ value of 266.8 nmole Pi update.

### Inhibition of sodium and phosphate absorption in rats

**Fig 5:**
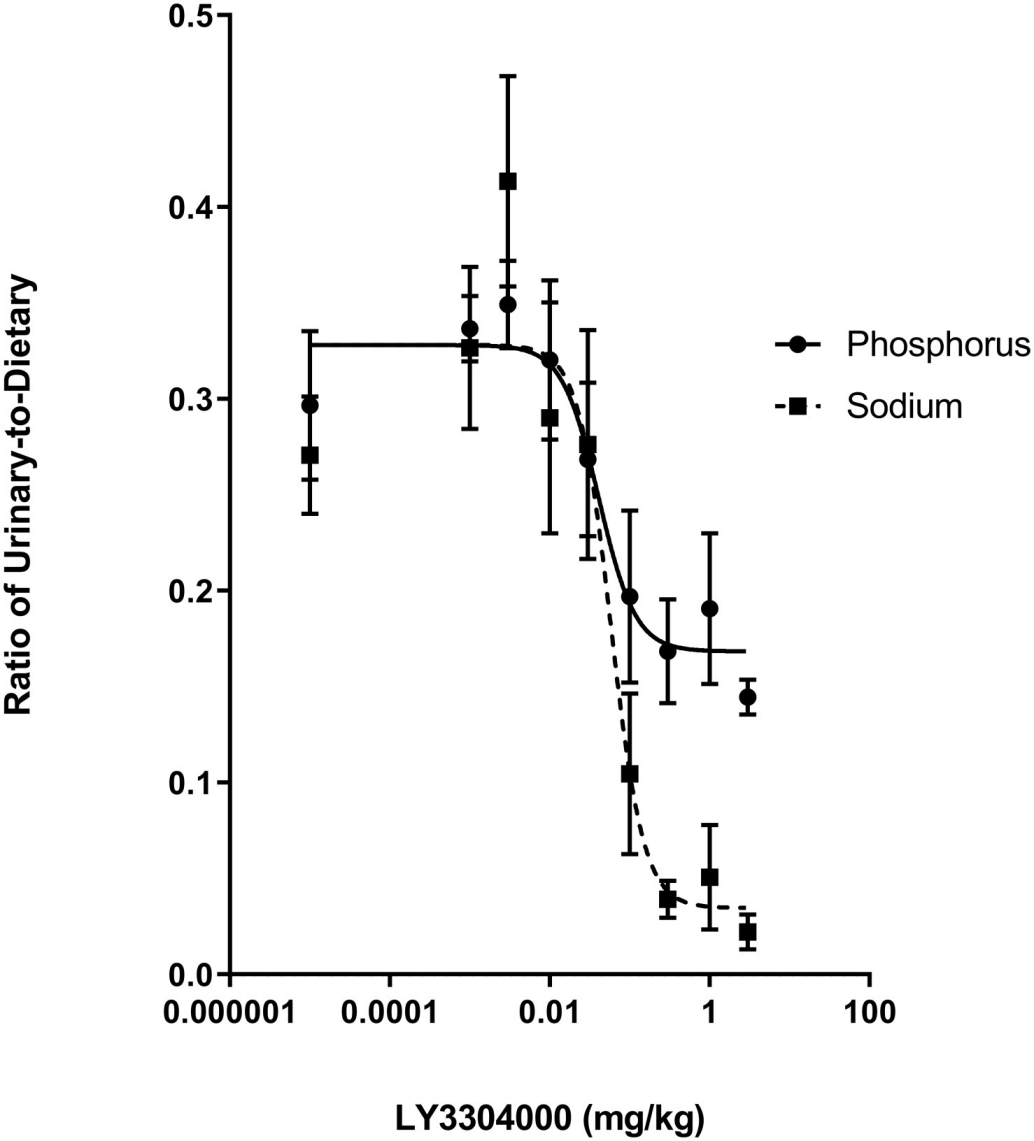
Effect of LY3304000 on sodium and phosphate (measured as phosphorus) excreted in urine in rats.

The apparent ability of LY3304000 to inhibit phosphate absorption was further explored in a rat model. Following an oral bolus of an NaH_2_PO_4_ solution to rats, urinary sodium and phosphate increased. In a dose dependent manner, LY3304000 suppressed the increase in both urinary sodium and phosphate (Fig 5 and S1 Table in S1 File). This is consistent with the NHE3 inhibitory activity of LY3304000 and the known ability of an intestinal NHE3 inhibitor to decrease intestinal sodium and phosphate absorption. The ED_50_ for the effect on sodium and phosphate were similar, consistent with both effects being due to an inhibition of NHE3.

Following treatment with an oral bolus of sodium phosphate and varying doses of LY3304000 in rats, urine was collected for 4 hours, then sodium, phosphorus, and creatinine appearing in urine were determined. Values expressed as the ratio of urinary phosphorus or sodium-to-dietary phosphorus, or sodium were calculated and presented as mean ± SEM. The curves were fitted with nonlinear regression with variable slope using GraphPad Prism to calculate the ED_50_. For the purpose of curve fitting, the vehicle was set to a dose of 0.00001 mg/kg. The slopes of the curve obtained from analysis are 1.90 and 2.13, for the urinary phosphate and sodium excretion, respectively. The ED_50_ values for the phosphorus and sodium uptake were 0.041 and 0.058 mg/kg, respectively.

### Effect of an NHE3 inhibitor used in combination with an NPT2b inhibitor on acute phosphate uptake into plasma of rats

**Fig 6:**
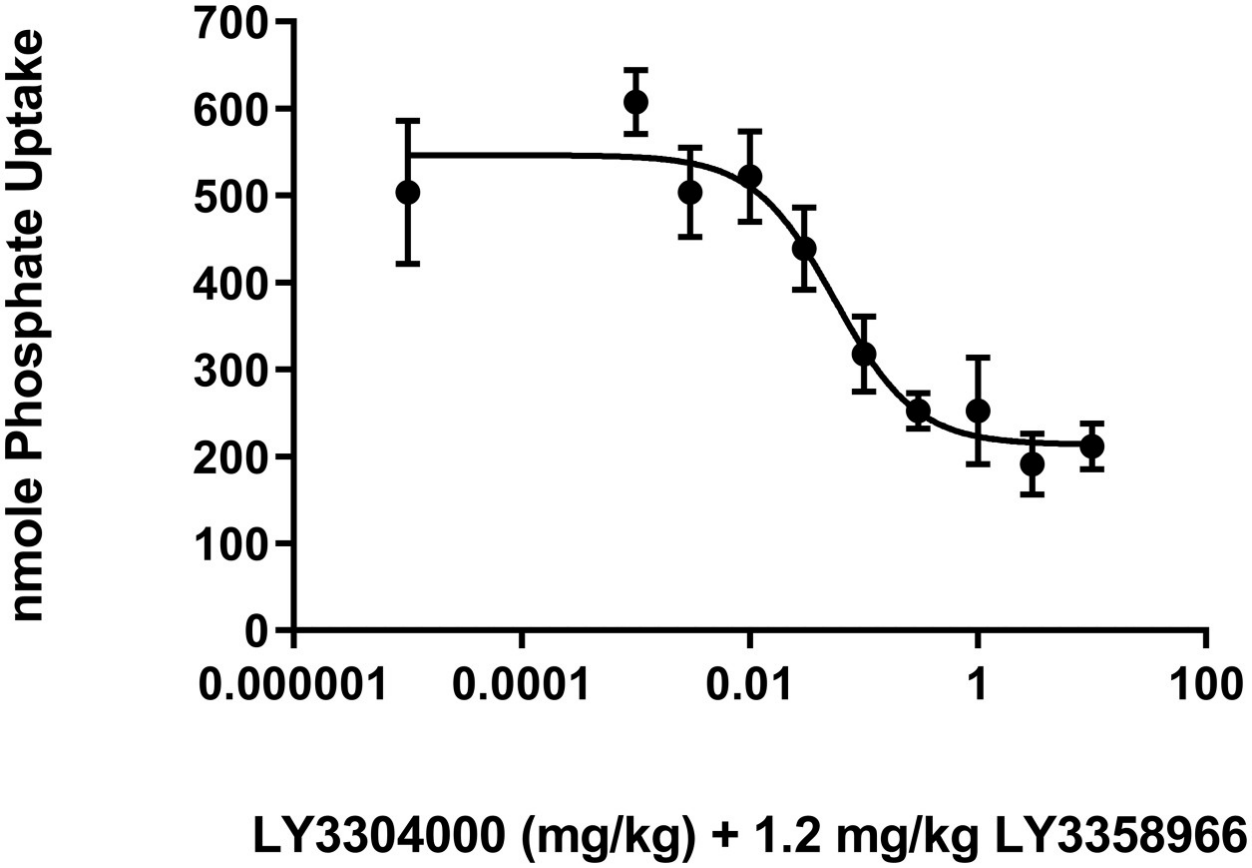
Acute phosphate uptake in rats treated with a fixed, 1.2 mg/kg dose of LY3358966 and increasing doses of LY3304000.

Fasted rats were administered vehicle, 1.2 mg/kg of the NPT2b inhibitor LY3358966, or increasing doses of LY3304000 in combination with a fixed, 1.2 mg/kg dose of LY3358966. The 1.2 mg/kg dose of LY3358966 was chosen for this study because it represents a dose that gives near maximal inhibition of phosphate absorption in rats [21]. Fifteen minutes later, the animals were dosed ^33^P phosphate. Another 15 minutes later, blood was collected, and the radioactivity in plasma was measured to calculate the radiolabeled phosphate recovered. The total radiolabeled phosphate recovered in plasma of the vehicle treated animals was 710 nmoles. Compared to the vehicle control, the NPT2b inhibitor, LY3358966, at a 1.2 mg/kg dose, showed only a trend towards a decrease in phosphate uptake to 504 nmoles or 71% of the vehicle control (Student’s t-Test, p = 0.15 versus vehicle control). When increasing doses of LY3304000 were combined with a fixed 1.2 mg/kg dose of LY3358966 (Fig 6), a further LY3304000 dose-dependent decrease in the acute phosphate uptake was observed with an ED_50_ of 0.056 mg/kg. At the highest dose tested, the acute phosphate uptake was decreased to 212 nmoles, a value 30% of vehicle control (i.e. 70% inhibition, Student’s t-Test, p = .002 versus vehicle control). This is identical to the maximal determined decrease based upon curve fitting. Thus, in rats, the NHE3 inhibitor, LY3304000, and NPT2b inhibitor, LY3358966, work in concert to inhibit acute phosphate uptake into plasma.

The absorption of radiolabeled phosphate measured at 15 minutes post dose was determined in rats. The phosphate uptake was defined as the percentage of administered radiolabeled phosphate recovered in the plasma multiplied by total amount of phosphate in Radiolabeled Phosphate Dosing Solution. Results are presented as mean ± SEM with animal numbers equal to 6 in the vehicle group and 5 or 6 in the compound treated groups. The curves were fitted with nonlinear regression with variable slope using GraphPad Prism. The slope of the curve obtained from analysis is 1.22. For the purposes of curve fitting, the 1.2 mg/kg LY3358966 alone was set to a dose of 0.00001 mg/kg of LY3304000. The ED50 value was calculated to be 0.056 mg/kg.

### Effect of an NHE3 inhibitor used in combination with an NPT2b inhibitor on phosphate absorption in rats

The effect of LY3304000 in combination with LY3358966 on phosphate absorption in rats was determined. Following the administration of compounds, ^33^P phosphate was dosed and then its recovery in plasma 15 minutes later (acute uptake), or in feces and the GI tract 4 hours later (absorption) were determined. Fasted rats were administered appropriate vehicles, 10 mg/kg LY3358966 (ED_50_ = 0.05 mg/kg in rats, [21]), 0.4 mg/kg LY3304000 (ED_50_ = 0.041 mg/kg in rats, Fig 4), or a combination of 0.4 mg/kg LY3304000 and 10 mg/kg LY3358966. These doses were chosen because they are doses that inhibit phosphate absorption to near E_max_ levels in acute rat assays.

**Fig 7:**
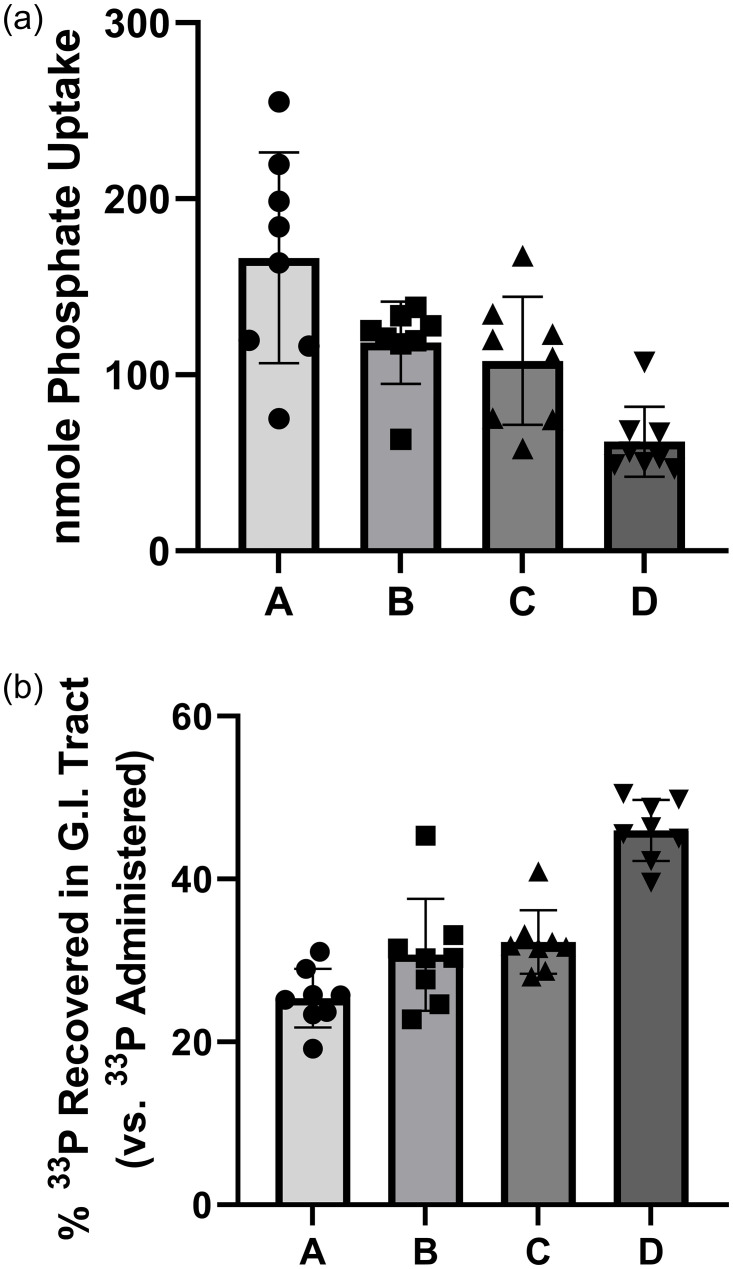
LY3304000, LY3358966, and an LY3304000/LY3358966 Combination Inhibited Radioactive Phosphate Absorption in Plasma (A) and Increased Its Recovery in the Gastrointestinal Tract (B) in Rats.

Fifteen minutes after dosing vehicle or compound, the animals were dosed with a ^33^P phosphate solution. Another 15 minutes later, blood was collected, and the acute phosphate uptake determined. While 0.4 mg/kg LY3304000 inhibited phosphate uptake by 29% (from 166 nmoles to 118 nmoles, p = 0.046 versus vehicle control) and 10 mg/kg LY3358966 inhibited phosphate absorption by 35% (from 166 nmoles to 108 nmoles, p = 0.013 versus vehicle control), the combination of the two compounds inhibited phosphate absorption by 63% (from 166 nmoles to 62 nmoles, p< 0.0001 versus vehicle control, Fig 7, panel A). The 63% inhibition by the 0.4 mg/kg LY3304000 and 10 mg/kg LY3358966 in combination exceeded the inhibition by either compound alone, though it barely missed significance compared to 10 mg/kg LY3358966 (p = 0.06).

The percent absorption of radiolabeled phosphate measured at 15 minutes in plasma was determined in rats (A). The mole amount of phosphate uptake into plasma was calculated as the percentage of administered radiolabeled phosphate recovered in the plasma multiplied by total amount of phosphate in Radiolabeled Phosphate Dosing Solution. Data were presented as mean ± SEM with animal numbers equal to 8 for the groups. Statistical significance was determined (p < 0.05) by a One-way ANOVA with a Dunnett’s comparison to the LY3304000/LY3358966 combination using GraphPad Prism. The mean ± SEM (*p*-value) for group vehicle, 0.4 mg/kg LY3304000, 10 mg/kg LY3358966 and LY3304000 + LY3358966 were 166 ± 21 (<0.0001), 118 ± 8 (0.02), 108 ± (0.06) and 62 ± 7 (NA) nmole, respectively. Mole amounts of phosphate uptake into plasma in Vehicle and LY3304000 or LY3358966 alone groups were compared to the LY3304000 and LY3358966 combination group using One-way ANOVA with a Dunnett’s comparison.

The percent dose recovered in GI tract was defined as radioactivity recovered in stomach, small intestine, large intestine, and feces compared to the amount administered (B). Data were presented as mean ± SEM with animal numbers equal to 8 for the groups. Statistical significance was determined by a Two-way ANOVA and Turkey HSD using JMP. The percentage of ^33^P recovery of group vehicle (i), 0.4 mg/kg LY3304000 (ii), 10 mg/kg LY3358966 (iii) and LY3304000 + LY3358966 (iv) were 25 ± 1, 31 ± 7, 32 ± 1 and 46 ± 1% respectively. The *p* value of comparison of group i vs ii, ii vs iii, iii vs i and (i, ii, or iii) vs iv were 0.132, 0.910, 0.032 and < 0.0001, respectively.

Four hours after the radiolabeled phosphate solution was dosed, stomach, small intestine, large intestine, and feces were collected then digested with 1N NaOH, and the recovered radioactivity was determined. In the vehicle treated rats, 25% of the dose was recovered (Fig 7, panel B and S2 Table in S1 File). Total radioactivity recovered was greater in animals dosed with both LY3304000 and LY3358966 (46%) than with either compound alone (31% or 32%, respectively). The percent dose recovered in GI tract data was further analyzed to test for additivity of the effects of LY3304000 and LY3358966 on inhibiting phosphate absorption. The test of LY3304000 and LY3358966 interaction was significant (p = 0.0187), indicating a synergistic relationship between the compounds at the doses tested in this study. That is, the inhibitory effect of the combination was greater than the sum of the individual compound effects.

### Effect of pH on the activity of NPT2b in vitro

**Fig 8:**
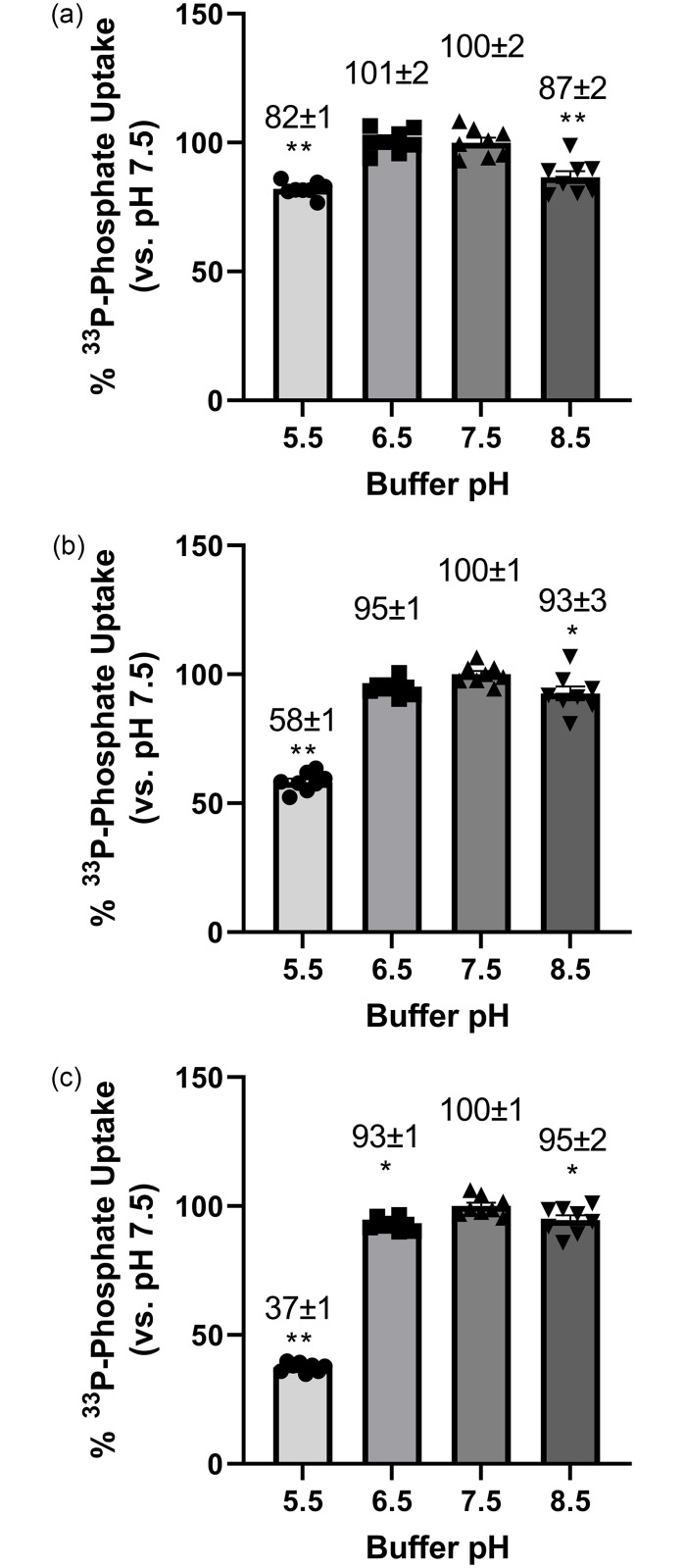
Effect of pH on Human (A), Mouse (B) or Rat (C) NPT2b Activity in Vitro.

Inhibition of NHE3 should increase the apical cell surface pH of intestinal epithelium. To determine whether this change in cell surface pH has the potential to affect NPT2b activity, we explored the effect of pH on NPT2b mediated phosphate uptake in vitro. The rate of phosphate uptake into CHO cells overexpressing human, mouse and rat NPT2b was measured as a function of assay buffer pH (Fig 8). As the pH of the buffer decreased below 6.5, the rate of NPT2b mediated phosphate uptake decreased, indicating NPT2b mediated phosphate transport is less effective at the lower pH.

^33^P-phosphate uptake was measured in CHO-TREX cells overexpressing human, mouse, or rat NPT2b in assay buffer with pH adjusted to 5.5, 6.5, 7.5, or 8.5. ^33^P-phosphate uptake was then normalized with the uptake in pH 7.5 buffer set to 100% and the results presented as mean ± SEM from 8 data points in single representative studies for human, rat and mouse NPT2b. * p-value < 0.05 and ** p-value < 0.0001 using ANOVA Dunnett’s multiple comparison test.

## Discussion

Balancing dietary phosphate absorption with renal excretion in patients with CKD and ESRD can be challenging. This has created a need for more effective therapies to inhibit the absorption of dietary phosphate, with the hope that this will better control the rise in serum phosphate, FGF23, PTH and decrease in 1,25-dihydroxyvitamin D, and the pathological sequela of their abnormal levels.

Dietary phosphate is absorbed in the intestine by paracellular diffusion and active transport, with more than one active transporter being implicated in the latter process [27–29]. Inhibiting one absorptive pathway apparently results in a compensating increase in another pathway, highlighting the challenge for controlling intestinal phosphate absorption and the phosphate burden in patients with renal disease. For example, we previously demonstrated that the NPT2b inhibitor, LY3358966, decreased the acute uptake of radiolabeled phosphate into the plasma of mice over 70%, however, over an extended period of time, it had minimal effect on phosphate absorption, suggesting other absorption pathways may be compensating for the loss of NPT2b activity [21]. Even when the NPT2b inhibitor was combined with a phosphate binder that decreases diffusional phosphate absorption, there was minimal effect on phosphate absorption. In contrast to phosphate binders which inhibit diffusional uptake of phosphate by decreasing the free phosphate levels in the intestinal lumen, inhibitors of the intestinal NHE3 are hypothesized to directly inhibit diffusional phosphate absorption by modulating the conformation of the intestinal tight junctions [16]. In these studies, we compared the effects of a novel NHE3 inhibitor on the acute uptake of phosphate in mice and rats, and further tested its ability to inhibit phosphate absorption in combination with a previously characterized NPT2b inhibitor.

Xue et al. [24] previously explored the role of intestinal NHE3 on acute phosphate uptake into plasma of mice. They developed a tamoxifen-inducible intestinal epithelial cell specific NHE3 knockout mouse model. Two weeks after tamoxifen induction, animals were dosed with ^33^P-phosphate at levels comparable to what we used in our studies. Plasma radioactive phosphate was lower in the NHE3 knockout mice than control mice 5 minutes after dosing with ^33^P phosphate, but the levels were similar at 15, 30 and 60 minutes. When a 60-fold higher dose of unlabeled phosphate was used in the study, there was a 1.8-fold higher increase in plasma phosphate in knockout versus control mice. Knockout mice compared to control mice had two-fold higher NPT2b protein levels in the small intestine and were reported to have greater intestinal permeability [30]. These changes may explain the increased phosphate absorption in this model, but they complicate our ability to understand the role of NHE3 in intestinal phosphate absorption.

LY3304000 is a potent NHE3 inhibitor in vitro that inhibits intestinal sodium absorption in mice and rats. When tested for its ability to affect the acute uptake of intestinal phosphate in mice, there was a robust two-fold increase in phosphate uptake from 39 nmoles to 78 nmoles, in contrast to the anticipated inhibition of uptake based upon previous studies in rats and humans [25, 26]. For comparison, following dosing with the NHE3 inhibitor, tenapanor, in a study similar to ours, Xue et al. observed ^33^P-phosphate levels in plasma were about 67% lower at 5 minutes post phosphate dose, compared to vehicle treated control animals. Plasma levels were similar at 15 and 30 minutes, but were significantly elevated about 50% at 60 min. One key difference between the studies is that we pre-dosed the animals with LY3304000 15 minutes before dosing radiolabeled phosphate, while Xue et al. co-administered tenapanor and phosphate [24]. In contrast, King et al. [16] found tenapanor had little effect on phosphate absorption using mouse ileum monolayer cultures and an in vivo ileum loop model.

As previously reported for tenapanor [26], LY3304000 modestly inhibits acute uptake of phosphate into plasma of rats (Fig 4) and inhibits phosphate absorption over time as indicated by the decreased urinary phosphate following dosing a bolus of sodium phosphate (Fig 5). To test the ability of LY3304000 to work in combination with an NPT2b inhibitor which modestly inhibits acute phosphate uptake in rats about 20–30% [21], increasing doses of LY3304000 were combined with a fixed 1.2 mg/kg dose of the NPT2b inhibitor LY3358966 (ED_50_ around 0.05 mg/kg in rats). Increasing doses of LY3304000 further decreased the acute uptake of phosphate into plasma beyond that of LY3358966 alone, indicating the two mechanisms work together to decrease phosphate uptake (Fig 6).

Compared to mice, rats appear to have a more human like profile for intestinal phosphate absorption including 1) modest NPT2b mediated phosphate absorption, 2) prominent expression of NPT2b in the proximal small intestine [20], and 3) an NHE3 inhibitor inhibits phosphate absorption. Because the rat model may more accurately translate to humans, we chose this model to characterize how and NHE3 inhibitor and NPT2b inhibitor, used in combination, affects phosphate absorption. Rats were dosed with LY3358966, LY3004000, or an LY3358966/LY3004000 combination. Four hours after dosing radiolabeled phosphate, the recovery of radiolabeled phosphate in the gastrointestinal tract of rats was measured. A four-hour time point was selected because most dietary phosphate is absorbed within four hours in rats and humans [23, 31–33], and our previous studies indicated the results in rats at four hours mirrored the recovery of phosphate in feces collected for 48 hours after dosing radiolabeled phosphate [21]. Neither NPT2b nor NHE3 inhibition alone produced a robust increase in the phosphate retained in the intestine compared to vehicle control (6–7%). However, when used in combination in rats, there was a clear synergist effect of the two inhibitors, achieving 21% more retention of phosphate than vehicle control. Importantly, in this study, each compound was dosed at a level at least 10 times the EC_50_ for the compound, a dose that produces near maximal effect when the compound is dosed alone in acute phosphate uptake studies in rats.

To help elucidate why NHE3 inhibition increases phosphate absorption in mice, we studied the effect of pH on NPT2b activity in vitro. Previous studies have reported conflicting findings for the effect of pH on NPT2b activity, with some studies finding less activity at lower pH [34], while others found little effect of pH on activity [35, 36]. We found rat and mouse NPT2b activity were higher at neutral pH compared to pH = 5.5 (Fig 8). As NHE3 transports protons from inside the intestinal epithelial cells into the intestinal lumen, the immediate cell surface pH should be more acidic than the intestinal lumen. An acidic microenvironment at the intestinal membrane surface has been confirmed experimentally [37, 38]. As NHE3 is inhibited, the apical cell surface pH should increase, resulting in increased NPT2b activity. This in part could explain why an NHE3 inhibitor increases, rather than decreases, acute intestinal phosphate uptake in mice, a model where acute phosphate uptake is dominated by NPT2b. Since the specificity of LY3304000 was not tested against other NHE found in the intestine epithelium, we cannot exclude that the inhibition of other NHEs could contribute to some extent to the observed effects. However, since NHE3 is the dominant NHE responsible for sodium absorption in the apical membrane of intestinal epithelial cells, it follows that its inhibition would dominate effects on pH at the cell surface [39]. Further support for the importance of NHE3 comes from the work of Xue et al. [24], They found that the NHE3 inhibitor tenapanor increased plasma ^33^P-phosphate 60 minutes after dosing ^33^P-phosphate in control mice, however, it had no effect in NHE3 knockout mice.

The effect of pH on NPT2b mediated phosphate transport may in part explain the synergistic effect between NHE3 and NPT2b inhibition in rats. Although not evident in rats where NHE3 inhibition decreases phosphate absorption by decreasing diffusional uptake, we speculate the level of decrease by NHE3 inhibition is diminished by a simultaneous increase in NPT2b mediated phosphate absorption. Thus, we hypothesize that combining an NPT2b inhibitor with an NHE3 inhibitors helps negate the NHE3 inhibitor mediated increase in NPT2b activity and produces a synergistic inhibition of phosphate absorption. Beyond cell surface pH, there may be other, yet unexplored ways in which NPT2b and NHE3 interact in the absorption of dietary phosphate. For example, NPT2b and NHE3 both associate with NHERF1 at the apical surface of enterocytes, and their cell surface expression is regulated by various factors, for example dietary phosphate for NPT2b or extracellular sodium for NHE3 [40, 41]. This raises the possibility that factors that affect the cell surface expression of one transporter may have an effect on the cell surface expression of the other, providing another layer of complexity for their interaction in phosphate absorption.

To be able to translate our finding to humans, a phosphate dose that was relevant to humans was selected (see materials and methods). By changing the phosphate dose, one could affect how phosphate absorption is distributed between active transport and passive diffusion. However, further studies in animal models using different phosphate doses would do little to further elucidate effects in humans. Because a human relevant phosphate dose was used in these studies, the qualitative conclusions of the studies should translate to humans, however, human studies are needed to fully understand quantitative effects. Even quantitative studies in humans are complicated by the variability between people and their diets.

In conclusion, previous studies in preclinical animal models and humans indicate NPT2b inhibitors as a monotherapy have a limited or no effect on intestinal phosphate absorption. NHE3 inhibitors at tolerated doses modestly inhibit phosphate absorption in rats and humans. Our studies performed in mice and rats produced conflicting findings for effects of NHE3 inhibition on acute intestinal phosphate uptake, decreasing acute uptake in rats and increasing it in mice. These studies further revealed a previously underappreciated complexity for how inhibitors of the different phosphate absorption pathways may interact. An NHE3 inhibitor combined with an NPT2b inhibitor synergistically inhibits phosphate absorption and may represent a novel therapeutic approach that addresses some of the shortcomings of targeted monotherapies used to date.

Although the results of our studies established that inhibition of NHE3 and NPT2b synergistically inhibit intestinal phosphate absorption in rats, there are several limitations of our studies that need to be recognized. First, we demonstrated LY3004000 inhibits NHE3, but we never determined its selectivity versus other NHEs, raising the possibility that inhibition of other intestinal NHEs could be contributing to some extent, the observed effects of LY3004000. Second, the mechanism of action for LY3004000 beyond its ability to inhibit NHE3 mediated proton transport was not explored. How closely our NHE3 inhibitor replicates the downstream effects reported for the NHE3 inhibitor, tenapanor [16], in the gastrointestinal tract has not been addressed. Third, although changes in cell surface pH with NHE3 inhibition provides a plausible explanation for the observed synergism between NHE3 and NPT2b inhibition, the extent of its contribution is not clear. Other unidentified factors may also be contributing to the observed synergism. Fourth, we anticipate the synergistic effect between NHE3 and NPT2b inhibition will translate to humans, however the magnitude of the effect in humans is unknown and can only be determined experimentally.

## Supporting information

S1 File (DOCX)

## Abbreviations

CKD: chronic kidney disease
ESRD: end-stage renal disease
NHE3: sodium hydrogen exchanger 3
NPT2b: sodium dependent phosphate cotransporter 2b
