# Supplementary material for: Effect of an NHE3 inhibitor in combination with an NPT2b inhibitor on gastrointestinal phosphate absorption in Rodent models
Source: PLoS One. 2024 Jan 26;19(1):e0292091. doi: 10.1371/journal.pone.0292091 (PMC10817170; doi:10.1371/journal.pone.0292091)
Supplement: S1 File — (DOCX) [file pone.0292091.s001.docx]

**Supplemental Materials**

**Supplemental Table 1: Effect of LY3304000 on Urinary Phosphorus (A) and Sodium (B) Excretion During 4 Hours in Rats**

**A.**

| Group | LY3304000 (mg/kg) | Urine Volume (mL) | SEM | Total Urinary Phosphorus (µg) | SEM | Phosphorus-to- Creatinine (mg/mg) | SEM | P value | Urinary  -to-  Dietary Phosphate | SEM | P value |
| --- | --- | --- | --- | --- | --- | --- | --- | --- | --- | --- | --- |
| 1 | N/A | 5.4 | 0.45 | 217 | 22.9 | 0.3 | 0.05 | .001 | N/A | N/A | N/A |
| 2 | N/A | 3.2 | 0.32 | 10799 | 1363 | 14.6 | 0.98 | N/A | 0.297 | 0.0388 | N/A |
| 3 | 0.001 | 4 | 0.52 | 12501 | 672 | 14 | 0.54 | 1 | 0.337 | 0.0171 | .95 |
| 4 | 0.003 | 4.6 | 0.48 | 12997 | 935 | 14.1 | 1.01 | 1 | 0.350 | 0.0229 | .83 |
| 5 | 0.01 | 3.8 | 0.68 | 12066 | 1665 | 15 | 0.94 | 1 | 0.320 | 0.0416 | 1 |
| 6 | 0.03 | 2.7 | 0.51 | 9961 | 1504 | 13 | 0.67 | .67 | 0.268 | 0.0401 | 1 |
| 7 | 0.1 | 1.7 | 0.39 | 7247 | 1608 | 12.3 | 0.64 | .29 | 0.197 | 0.0449 | .21 |
| 8 | 0.3 | 1 | 0.16 | 6250 | 1029 | 12 | 0.55 | .2 | 0.168 | 0.0271 | .056 |
| 9 | 1 | 1.4 | 0.20 | 7081 | 1489 | 11.3 | 1.50 | .0369 | 0.191 | 0.0392 | .16 |
| 10 | 3 | 1 | 0.10 | 5346 | 378 | 9.5 | 0.23 | .0006 | 0.144 | 0.0090 | .0216 |

**B.**

| Group | LY3304000 (mg/kg) | Urine Volume (mL) | SEM | Total Urinary Sodium (µg) | SEM | Sodium  -to- Creatinine (mg/mg) | SEM | P value | Urinary-to-  Dietary Sodium | SEM | P value |
| --- | --- | --- | --- | --- | --- | --- | --- | --- | --- | --- | --- |
| 1 | N/A | 5.4 | 0.45 | 2858 | 536 | 3.9 | 0.72 | .0007 | N/A | N/A | N/A |
| 2 | N/A | 3.2 | 0.32 | 7363 | 823 | 10.2 | 1.11 | N/A | 0.271 | 0.0306 | N/A |
| 3 | 0.001 | 4 | 0.52 | 9084 | 1255 | 10 | 1.07 | 1 | 0.326 | 0.0423 | .92 |
| 4 | 0.003 | 4.6 | 0.48 | 11429 | 1493 | 12.2 | 1.33 | .768 | 0.413 | 0.0549 | .112 |
| 5 | 0.01 | 3.8 | 0.68 | 8104 | 1685 | 9.8 | 1.27 | 1 | 0.290 | 0.0602 | .999 |
| 6 | 0.03 | 2.7 | 0.51 | 7637 | 1674 | 9.8 | 1.52 | 1 | 0.276 | 0.0598 | 1 |
| 7 | 0.1 | 1.7 | 0.39 | 2850 | 1100 | 5.3 | 1.27 | .0163 | 0.105 | 0.0418 | .0436 |
| 8 | 0.3 | 1 | 0.16 | 1095 | 279 | 2.4 | 0.80 | <.0001 | 0.039 | 0.0097 | .0035 |
| 9 | 1 | 1.4 | 0.20 | 1400 | 756 | 2.4 | 0.99 | <.0001 | 0.051 | 0.0271 | .0019 |
| 10 | 3 | 1 | 0.10 | 612 | 253 | 1 | 0.38 | <.0001 | 0.022 | 0.0091 | .0013 |

**Supplemental Table 2: The Percentage of Radiolabeled Phosphate Recovered in Each Section of the Gastrointestinal Tract**

| Treatment | Stomach | Small Intestine | Large Intestine | Feces | Total |
| --- | --- | --- | --- | --- | --- |
| Control-PVP + HEC | 5.50 +/- 0.89 | 8.03 +/- 0.54 | 11.83 +/- 1.09 | <0.1 | 25.36 +/- 1.27 |
| LY3304000, 0.4 mg/kg | 3.9 +/- 0.49 | 8.64 +/- 0.32 | 18.16 +/- 2.58 | <0.1 | 30.7 +/- 2.43 |
| LY3358966, 10 mg/kg | 4.86 +/- 1.13 | 8.98 +/- 0.48 | 18.43 +/- 2.03 | <0.1 | 32.27 +/- 1.38 |
| LY3304000 + LY3358966 | 6.23 +/- 0.80 | 10.76 +/- 0.67 | 28.98 +/- 1.93 | <0.1 | 45.96 +/- 1.33 |

**Supplemental Table 3: The Comparison of LY3304000 Properties with the Preferred Properties of Orally Bioavailable Compounds***

| Preferred properties of orally bioavailable compounds | LY3304000 Properties |
| --- | --- |
| Molecular mass less than 500 Da | **1302** |
| cLogP ≤ 5 | **4.14 (Chemaxon)** |
| No more than 5 hydrogen bond donors | **10 H-bond donors** |
| No more than 10 hydrogen bond acceptors | **22 H-bond acceptors** |
| 10 or fewer rotatable bonds | **31** |
| Polar surface area no greater than 140 Å2 | **312 Å^2^** |

* Lipinski et al. (2001) Adv Drug Delivery Rev 46: 3-26 and Zakeri-Milani et al. (2006) DARU 14: 164-171.

**Scheme 1**

**General Experimental**

Racemic 3-[6,8-dichloro-2-methyl-3,4-dihydro-1H-isoquinolin-4-yl]benzenesulfonyl chloride was purchased from Wuxi PharmaTech. All reagents were purchased from commercial vendors and used without any further manipulation. LC-ES/MS analysis was performed on an Agilent HP1100 liquid chromatography system. Electrospray mass spectrometry measurements (acquired in positive and/or negative mode) were performed on a Mass Selective Detector quadrupole mass spectrometer that was interfaced to the HP1100 HPLC. NMR spectra were obtained on a Bruker AVIII HD 400 MHz NMR Spectrometer or a Varian VNMRS 300 or 400 MHz NMR Spectrometer. Chemical shifts are reported in parts per million and referenced to CDCl_3_ (7.26 ppm for ^1^H) or DMSO-d_6_ (2.50 ppm for ^1^H) as internal standard. Peak multiplicities are reported as s (singlet), d (doublet), t (triplet), q (quartet), m (multiplet), br s (broad singlet), dd (doublet of doublets) and dt (doublet of triplets). Coupling constants (*J*) are reported in hertz (Hz).

**tert-Butyl N-[2-[2-[2-[[3-[(4S)-6,8-dichloro-2-methyl-3,4-dihydro-1H-isoquinolin-4-yl]phenyl]sulfonylamino]ethoxy]ethoxy]ethyl]carbamate (2).** To a solution of tert-butyl N-[2-[2-(2-aminoethoxy)ethoxy]ethyl]carbamate (5.22 g, 21.0 mmol) and trimethylamine (8.79 mL, 63.0 mmol, 3.00 equiv.) in anhydrous dichloromethane (120 mL) was added racemic 3-[6,8-dichloro-2-methyl-3,4-dihydro-1H-isoquinolin-4-yl]benzenesulfonyl chloride (8.21 g, 21.0 mmol) and stirred at room temperature under nitrogen overnight. After removing the volatiles under reduced pressure, ethyl acetate (50 mL) was added, and solids were filtered off. The resulting solution was concentrated under reduced pressure to afford an orange oil, which was purified by silica gel column chromatography eluting with ethyl acetate to afford the title compound as a clear oil (6.92 g, 55%). ES/MS m/z (^35^Cl/^37^Cl) 602.4.0/604.4 [M+H]^+^. Enantiomers were separated on a Chiralpak AD (8 x 35 cm) column (flow rate 400 mL/min, detection at 260 nm) eluting with 95:5 ethanol:acetonitrile to obtain 3.54 g of isomer 1 and 2.94 g of isomer 2 (S isomer, desired). Analytical conditions: Column: Chiralpak AD-H (4.6 x 150 mm). Flow: 0.6 mL/min**.** Detection: 250 nm**.** Eluent: 95:5 ethanol:acetonitrile. Isomer 1 (R isomer): retention time 3.87 min, 94.2% enantiomeric excess. Isomer 2 (S isomer): retention time 5.94 min, >99% enantiomeric excess.

**N-[2-[2-(2-aminoethoxy)ethoxy]ethyl]-3-[(4S)-6,8-dichloro-2-methyl-3,4-dihydro-1H-isoquinolin-4-yl]benzenesulfonamide (3).** To tert-butyl N-[2-[2-[2-[[3-[(4S)-6,8-dichloro-2-methyl-3,4-dihydro-1H-isoquinolin-4-yl]phenyl]sulfonylamino]ethoxy]ethoxy]ethyl]carbamate (2.94 g, 4.88 mmol) was added 4M hydrogen chloride in dioxane (10 mL) and the resulting solution was stirred at room temperature overnight. Volatiles were removed under reduced pressure to afford a white foam (2.85 g, >100%), that was further dried under high vacuum overnight and used without further manipulation. ES/MS m/z (^35^Cl/^37^Cl) 502.0/504.0 [M+H]^+^**.** A portion of crude product (992 mg, 1.84 mmol) was purified using a 10 g Isolut SCX column to obtain 740 mg of the free base as a clear colorless semi-solid (740 mg, 80%).

**2-cyano-1-[4-[[(Z)-N'-cyano-N-[2-[2-[2-[[3-[(4S)-6,8-dichloro-2-methyl-3,4-dihydro-1H-isoquinolin-4-yl]phenyl]sulfonylamino]ethoxy]ethoxy]ethyl]carbamimidoyl]amino]butyl]-3-[2-[2-[2-[[3-[(4S)-6,8-dichloro-2-methyl-3,4-dihydro-1H-isoquinolin-4-yl]phenyl]sulfonylamino]ethoxy]ethoxy]ethyl]guanidine (4).** To a solution of N-[2-[2-(2-aminoethoxy)ethoxy]ethyl]-3-[(4S)-6,8-dichloro-2-methyl-3,4-dihydro-1H-isoquinolin-4-yl]benzenesulfonamide (740 mg, 1.473 mmol) in dioxane (10 mL) and pyridine (10 mL) was added diphenoxymethylenecyanamide (351 mg, 1.47 mmol) and stirred at room temperature under nitrogen overnight. Butane-1,4-diamine (65 mg, 0.7374 mmol) was added, and the reaction was heated at 60°C overnight. It was then heated at 90°C overnight. After cooling to room temperature, the reaction was diluted with ethyl acetate, washed with 5% aqueous potassium carbonate twice, dry over anhydrous magnesium sulfate, filtered and concentrated under reduced pressure to yield an orange oil (1.08 g). The crude product was purified by silica gel column chromatography eluting with a 0 to 10% methanol in dichloromethane gradient to afford the title compound as a yellow foam (591 mg, 67%). ES/MS m/z (^35^Cl) 1193.4 [M+H]^+^.

**(Z)-[[4-[[(Z)-N'-carbamoyl-N-[2-[2-[2-[[3-[(4S)-6,8-dichloro-2-methyl-3,4-dihydro-1H-isoquinolin-4-yl]phenyl]sulfonylamino]ethoxy]ethoxy]ethyl]carbamimidoyl]amino]butylamino]-[2-[2-[2-[[3-[(4S)-6,8-dichloro-2-methyl-3,4-dihydro-1H-isoquinolin-4-yl]phenyl]sulfonylamino]ethoxy]ethoxy]ethylamino]methylene]urea (5).** To 2-cyano-1-[4-[[(Z)-N'-cyano-N-[2-[2-[2-[[3-[(4S)-6,8-dichloro-2-methyl-3,4-dihydro-1H-isoquinolin-4-yl]phenyl]sulfonylamino]ethoxy]ethoxy]ethyl]carbamimidoyl]amino]butyl]-3-[2-[2-[2-[[3-[(4S)-6,8-dichloro-2-methyl-3,4-dihydro-1H-isoquinolin-4-yl]phenyl]sulfonylamino]ethoxy]ethoxy]ethyl]guanidine (670 mg, 0.562 mmol) was added trifluoroacetic acid (10 mL) and water (1 mL) and the reaction was stirred at room temperature overnight. After remove volatiles under reduced pressure, the residue was dissolved in a minimum amount of methanol and purified through a 10 g Isolut SCX column to afford a yellow foam (629 mg). The product was further purified by preparative high performance liquid chromatography (high pH) to obtain the title compound as an orange oil (253 mg, 37%). ES/MS m/z (^35^Cl) 1229.2 [M+H]^+^.

**(Z)-[[4-[[(Z)-N'-carbamoyl-N-[2-[2-[2-[[3-[(4S)-6,8-dichloro-2-methyl-3,4-dihydro-1H-isoquinolin-4-yl]phenyl]sulfonylamino]ethoxy]ethoxy]ethyl]carbamimidoyl]amino]butylamino]-[2-[2-[2-[[3-[(4S)-6,8-dichloro-2-methyl-3,4-dihydro-1H-isoquinolin-4-yl]phenyl]sulfonylamino]ethoxy]ethoxy]ethylamino]methylene]urea dihydrochloride, LY3304000 (2).** In a vial, to a solution of (Z)-[[4-[[(Z)-N'-carbamoyl-N-[2-[2-[2-[[3-[(4S)-6,8-dichloro-2-methyl-3,4-dihydro-1H-isoquinolin-4-yl]phenyl]sulfonylamino]ethoxy]ethoxy]ethyl]carbamimidoyl]amino]butylamino]-[2-[2-[2-[[3-[(4S)-6,8-dichloro-2-methyl-3,4-dihydro-1H-isoquinolin-4-yl]phenyl]sulfonylamino]ethoxy]ethoxy]ethylamino]methylene]urea (223 mg, 0.181 mmol) in dichloromethane (0.5 mL) was added hydrochloric acid (1M solution in diethyl ether, 1 mL) drop wise with shaking. White solids crushed out of solution. The mixture was shaken for five minutes and the volatiles were evaporated under reduced pressure to afford the title compound as a fine white powder (236 mg, 100%). ES/MS m/z (^35^Cl) 1229.4 [M+H]^+^.

**Characterization of the cell permeability of LY3304000**

LY3304000 has very low in vitro cell permeability. In a standard Madin-Darby Canine Kidney (MDCK) cell uni-directional permeability assay, measuring the percentage A to B (% A-B) transport in a 24-well format, the % A-B of LY330400 transport is **0.16 %** at 20 μM for the assay duration of one hour.

**Pharmacokinetics Study of LY3304000 in Rats**

Following oral dosing of the free base from of LY3304000 in a vehicle of 1% Hydroxyethylcellulose, 0.25% Polysorbate 80, and 0.05% Antifoam in purified water, the level of LY3304000 in rat blood 2.5-hour post dose was below the limit of quantification of LY330400 (0.815 nM with LC/MS method). Further characterization of the pharmacokinetic properties of LY3304000 was hampered by an inability to identify an appropriate vehicle for IV dosing.
